# Complete Genome Sequence of an Avian Reovirus Isolated from Wild Mallard Ducks in China

**DOI:** 10.1128/genomeA.00813-14

**Published:** 2014-09-18

**Authors:** Kexiang Yu, Yufeng Li, Hongyu Han, Minxun Song, Xiuli Ma, Cunxia Liu, Bin Huang, Feng Li

**Affiliations:** a Institute of Poultry, Shandong Academy of Agricultural Sciences, Key Laboratory of Poultry Disease Diagnosis and Immuonology of Shandong Province, Jinan, China; b College of Animal Science & Veterinary Medicine, Shandong Agricultural University, Tai’an, China; c Department of Veterinary and Biomedical Sciences, South Dakota State University, Brookings, South Dakota, USA; d Department of Biology and Microbiology, South Dakota State University, Brookings, South Dakota, USA

## Abstract

We report here the complete sequence of novel duck reovirus (N-DRV) strain SD12 isolated from diseased wild mallard ducklings in the Shandong Province of China in 2012. The complete genome consists of 23,420 nucleotide base pairs (bp), including 10 segments ranging from 1,191 bp (S4) to 3,959 bp (L1).

## GENOME ANNOUNCEMENT

Avian orthoreovirus (ARV) is a member of the genus *Orthoreovirus* in the family *Reoviridae* that causes, most importantly, arthritis and tenosynovitis in poultry including chickens, turkeys, ducks, and geese (1, 2). Causative ARVs consist of the early Muscovy duck reovirus, termed ARV-Md, and the newly emerged pathotype of Muscovy duck origin reovirus, termed N-MDRV (3, 4). These two virus types are antigenically different (5).

The genome of ARVs consists of 10 double-stranded RNA segments that can be further separated into three classes in size by their electrophoretic mobility: large (L1, L2, L3), medium (M1, M2, M3), and small (S1, S2, S3, S4) (6).

To date, ARV has not been isolated from wild duck species including mallard ducks. Here we report the complete genome of an ARV isolated from diseased wild mallard ducklings in the Shandong province, China. Following the initial positive diagnosis of ARV infection by real-time reverse transcription-PCR, we determined the full-length genome of the virus isolate using Sanger sequencing technology. The 5′ terminal nucleotide sequences of the genome segments were determined by a 5′ rapid amplification of cDNA ends protocol (5′ RACE). BLASTp searches of the nucleotide sequences and putative proteins identified that the virus belongs to the novel Muscovy duck origin reovirus. Consequently, the virus was provisionally designated N-DRV strain SD12.

The complete genome of N-DRV strain SD12 is 23,420 nucleotide base pairs (bp) long, and the sizes of the 10 segments are as follows: L1, 3,959 bp; L2, 3,830 bp; L3, 3,907 bp; M1, 2,284 bp; M2, 2,158 bp; M3, 1,996 bp; S1, 1,569 bp; S2, 1,324 bp; S3, 1,202 bp; and S4, 1,191 bp. With the exception of the S1 segment, which has three open reading frames (ORFs), all the genome segments have only one ORF, encoding corresponding primary polyproteins. The ORFs of S1 are partially overlapped and three putative proteins are produced: p10 with 97 amino acids (aa); p18 with 162 aa; σC with 321 aa. The sizes of the proteins encoded by the other 9 segments are as follows: λA, 1293 aa; λB, 1259 aa; λC, 1285 aa; μA, 732 aa; μB, 675 aa; μNS, 635 aa; σA, 416 aa; σB, 367 aa; and σNS, 367 aa.

All 10 segments or 12 putative proteins of SD12, respectively, had a greater than 94% identity in the nucleotide sequences or 98% identity in the amino acid sequences to corresponding counterparts of a DRV strain 091 isolated from Pekin ducklings in China in 2009 (7). The high similarity indicated that the two strains are the same virus. This result suggests host range expansion of N-DRV, although it is not clear how this transmission between wild mallard and domestic Pekin ducks occurs. Introduction of this pathogen to wild ducks highlights a need of further investigation and better surveillance of poultry flocks and birds.

### Nucleotide sequence accession numbers.

The sequences of the 10 segments of N-DRV strain SD12 have been deposited in GenBank under accession numbers KJ879924 (segment L1) to KJ879933 (segment S4).

## References

[1] Jones RC Guneratne JR 1984 The pathogenicity of some avian reoviruses with particular reference to tenosynovitis. Avian Pathol. 13:173–189. 10.1080/03079458408418522 18766835

[2] Zhang Y Guo D Geng H Liu M Hu Q Wang J Tong G Kong X Liu N Liu C 2007 Characterization of M-class genome segments of muscovy duck reovirus S14. Virus Res. 125:42–53. 10.1016/j.virusres.2006.12.004 17218035

[3] Yun T Yu B Ni Z Ye W Chen L Hua J Zhang C 2013 Isolation and genomic characterization of a classical Muscovy duck reovirus isolated in Zhejiang, China. Infect. Genet. Evol. 20:444–453. 10.1016/j.meegid.2013.10.004 24140560

[4] Chen S Chen S Lin F Wang S Jiang B Chen X Zhu X Zhang S 2012 The isolation and identification of novel duck reovirus. Chin. J. Virol. 28:224–230 22764524

[5] Chen S Chen S Cheng X Jiang B Lin F Wang S Zhu X Li Z Zhang S 2011 The comparison of serologic relativity and CPE type of 3 avian reovirus strains induced different diseases. Acta Vet. Zootech. Sin. 42:533–537

[6] Benavente J Martínez-Costas J 2007 Avian reovirus: structure and biology. Virus Res. 123:105–119. 10.1016/j.virusres.2006.09.005 17018239

[7] Ma G Wang D Shi J Jiang T Yuan Y Zhang D 2012 Complete genomic sequence of a reovirus isolate from Pekin ducklings in China. J. Virol. 86(23):13137. 10.1128/JVI.02512-12 23118462 PMC3497648

